# Mobile tablet-based therapies following stroke: A systematic scoping review of administrative methods and patient experiences

**DOI:** 10.1371/journal.pone.0191566

**Published:** 2018-01-23

**Authors:** Michael Pugliese, Tim Ramsay, Dylan Johnson, Dar Dowlatshahi

**Affiliations:** 1 School of Epidemiology and Public Health, University of Ottawa, Ottawa, Ontario, Canada; 2 Clinical Epidemiology Program, Ottawa Hospital Research Institute, Ottawa, Ontario, Canada; 3 Neuroscience Program, Ottawa Hospital Research Institute, Ottawa, Ontario, Canada; 4 Medicine (Neurology), Ottawa Hospital, Ottawa, Ontario, Canada; University of Tennessee Health Science Center, UNITED STATES

## Abstract

**Background and purpose:**

Stroke survivors are often left with deficits requiring rehabilitation to recover function and yet, many are unable to access rehabilitative therapies. Mobile tablet-based therapies (MTBTs) may be a resource-efficient means of improving access to timely rehabilitation. It is unclear what MTBTs have been attempted following stroke, how they were administered, and how patients experienced the therapies. The review summarizes studies of MTBTs following stroke in terms of administrative methods and patient experiences to inform treatment feasibility.

**Methods:**

Articles were eligible if they reported the results of an MTBT attempted with stroke participants. Six research databases were searched along with grey literature sources, trial registries, and article references. Intervention administration details and patient experiences were summarized.

**Results:**

The search returned 903 articles of which 23 were eligible for inclusion. Most studies were small, observational, and enrolled chronic stroke patients. Interventions commonly targeted communication, cognition, or fine-motor skills. Therapies tended to be personalized based on patient deficits using commercially available applications. The complexity of therapy instructions, fine-motor requirements, and unreliability of internet or cellular connections were identified as common barriers to tablet-based care.

**Conclusions:**

Stroke patients responded positively to MTBTs in both the inpatient and home settings. However, some support from therapists or caregivers may be required for patients to overcome barriers to care. Feasibility studies should continue to identify the administrative methods that minimize barriers to care and maximize patient adherence to prescribed therapy regiments.

## Background

### Rationale

Stroke survivors experience many post-stroke complications limiting their ability to function independently, including limitations to mobility [1], upper-limb function [2], communication [3], and cognition [4]. Specialized stroke rehabilitation has been shown to improve functional independence better than non-specialized care [5], and is most effective when performed early [6,7] and intensely post-stroke [8]. However, due to the growing number of stroke survivors [9] and lack of rehabilitation resources [10–13], many patients are not able to begin rehabilitative therapies early in order to maximize functional recovery. Easily accessible and resource-efficient stroke rehabilitation is needed to provide all stroke survivors with the opportunity to recover their functional abilities and improve their independence.

Mobile tablet computers with therapeutic applications could potentially offer a means of providing early and resource-efficient stroke rehabilitation. Tablet computers can be easily purchased and there exist many inexpensive or free therapy applications with scenarios analogous to those used in stroke rehabilitation. These devices are portable with fairly large, touch-responsive screens, which could be manipulated by stroke survivors depending on their post-stroke disabilities. Rehabilitation therapists can prescribe and monitor specific applications to patients based on their post-stroke difficulties.

Mobile tablet-based therapies (MTBTs) following stroke are an exciting new paradigm with many possibilities. However much remains unclear about this approach including the suitable population, the appropriate timing and setting, the necessary patient-support structure, and general therapy adherence. To better understand the feasibility of this intervention, it is critical to understand previous approaches to MTBT administration and the resulting patient experience, including treatment barriers and therapy adherence. A thorough review of the literature may provide important information for the successful conduct of large and potentially costly randomized controlled studies designed to demonstrate treatment efficacy.

### Objective

The objective of this study was to summarize the administration of mobile tablet-based therapy (MTBTs) following stroke and the subsequent patient experience to inform treatment feasibility. In particular, we sought to accomplish this objective by answering three research questions:

1. What post-stroke deficits or complications have been targeted by MTBTs and how were the therapies administered?
2. What barriers to care did patients experience while engaging in MTBTs following stroke?
3. Were patients adherent to MTBTs following stroke?

## Methods

### Protocol and registration

The design of this scoping review was guided by the most current and widely known guide for scoping reviews [14], and by PRISMA-P [15] and PRISMA where applicable [16]. PROSPERO currently does not accept scoping review protocols for registration and therefore the study protocol was hosted on the University of Ottawa research depository and can be accessed here: http://hdl.handle.net/10393/35696 (S1 Protocol).

### Eligibility criteria

All included articles were required to meet the following criteria: (1) the study population includes adult stroke survivors (18 years or older) of any type (ischemic/hemorrhagic) or stage (acute/chronic) in any setting, and (2) the study intervention involves stroke survivors interacting with a mobile tablet in response to a post-stroke deficit or complication. Articles were excluded if they met one or more of the following criteria: (1) the mobile tablet is primarily used by someone other than the stroke survivor, (2) the mobile tablet is more correctly described as an E-reader, and (3) the manuscript is a study protocol or conference abstract containing data otherwise available from a full study manuscript.

We defined MTBTs as patient-driven interactions with mobile tablets via various modalities for therapeutic purposes in response to a deficit, complication, or in order to prevent further health deterioration. There were no restrictions with regards to comparators, outcomes, study design and context, or settings, and conference abstracts were included if no full study manuscript could be found. However, only articles written in English were included and the search was limited to articles written from 2010 onwards as mobile tablets did not become widely popular until this time.

### Information sources

Six databases were searched: MEDLINE (OVID interface), EMBASE (OVID interface), PsycINFO (OVID interface), CINAHL, Cochrane Database, and Web of Science. Additional sources were used to augment the database search for academic material: (1) a snowball search of relevant articles and reviews identified by the database search, (2) stroke research-related organizational websites, and (3) clinical trial databases were searched for completed and ongoing studies. A grey literature search was performed to find unpublished material using Google Scholar, the ProQuest Dissertation and Theses Database (Global and UK & Ireland), and the OpenGrey European grey literature database.

### Search: Medline (Ovid interface) search strategy

1. exp Stroke/
2. exp cerebrovascular disorders/
3. (stroke* or cerebrovascular* or cerebral vascular or CVA*).tw.
4. ((cerebr* or brain) adj3 infarct*).tw.
5. 1 or 2 or 3 or 4
6. (mobile device* or mobile computer* or handheld computer* or tablet*).tw.
7. (ipad* or galaxy tab* or surface pro*).tw.
8. 6 or 7
9. 5 and 8
10. Restrictions: published in English between 2010-Present.

### Study selection

A two-stage screening process performed by two independent reviewers was used to select included studies. In stage one, two authors (MP, DJ) screened abstracts and titles for potentially relevant articles, and in stage two, two authors (MP, DJ) read potentially relevant articles to confirm they met the inclusion criteria.

### Data collection and data items

Two authors (MP, DJ) independently extracted data with the assistance of a data extraction form (S2 Appendix). Data items were selected based on the research objective and questions stated above. Items included general study information, participant characteristics, intervention details, comparator description, study outcome description and results, study setting and other contextual information.

### Risk of bias in individual studies

As per current guidelines, no risk of bias assessment was performed for individual studies [14].

### Analyses

The review goals and content were not appropriate for quantitative synthesis techniques commonly used in meta-analyses of systematic reviews. However, general study characteristics, participants, interventions, comparators, and patient-reported experiences were summarized narratively and using descriptive statistics where appropriate.

## Results

### Study selection

**Fig 1:**
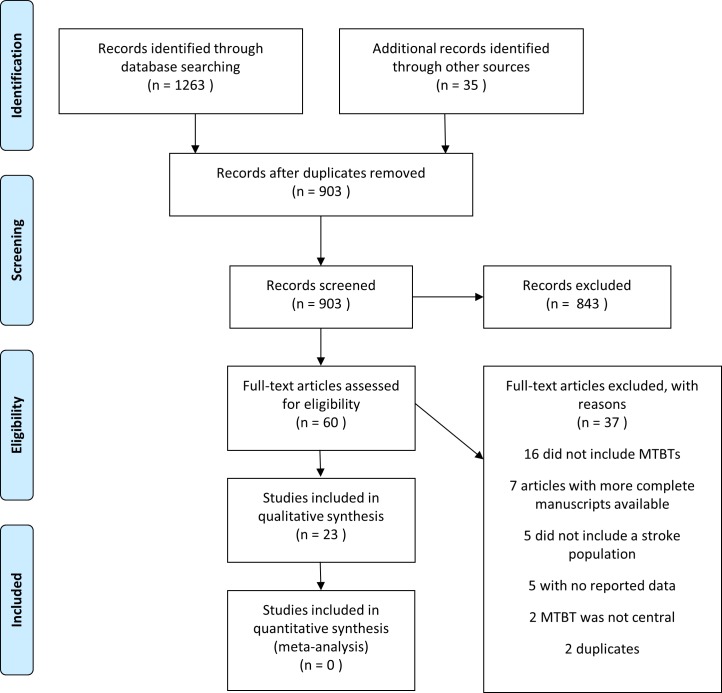
PRISMA flow diagram.

The search returned 868 articles from database searches, 8 potentially relevant articles from grey literature searches, 11 unique clinical trials from registry searches, and 16 potentially relevant articles through snowball reference searches of included studies for a total of 903 articles (Fig 1). Title and abstract screening narrowed the search down to 60 articles of which 37 were excluded leaving 23 articles for inclusion [17–39]. The eligible articles came from various search sources; 16 from database searches, 2 from grey literature, 2 from snowball searches, and included both full-texts and abstracts. Seven of the included articles were abstracts, the remaining articles were full manuscripts. None of the clinical trials identified by registry searches provided preliminary data or were completed, and therefore were not formally included.

### Study characteristics

Most of the included studies were observational; 10 cohort studies, 3 case studies, 2 cross-sectional studies, and 1 qualitative interview (Table 1). Of the 7 experimental studies, 3 were RCTs, 2 were randomized experiments, 1 was a non-randomized experiment, and 1 used a cross-over design. Sample sizes were small ranging from 1 to 63 with an average of 18 participants. Three articles (Salaris et al. 2016 (1), Salaris et al. 2016 (2), and Janssen et al. 2016) were conference abstracts based off the same “TnT” study, although they reported different results.

**Table 1:** Studies of mobile tablet-based therapies following stroke.

Article	Format	Design	Stroke Stage	Sample	Tablet Experience*
Carabeo et al. 2014	manuscript	cohort	chronic	3	1
Choi et al. 2016 (1)	manuscript	cohort	chronic	8	7
Choi et al. 2016 (2)	manuscript	experiment	subacute	24	not reported
Crotty et al. 2014	manuscript	cohort	not reported	32	not reported
Davis & Holzbach 2014	abstract	case study	not reported	1	not reported
Des Roches et al. 2015	manuscript	experiment	subacute/chronic	51	22
Hiyamizu et al. 2013	abstract	experiment	unclear	10	not reported
Hoover & Carney 2014	manuscript	cohort	chronic	20	5
Jang & Jang 2016	manuscript	experiment	chronic	21	not reported
Janssen et al. 2016	abstract	experiment	not reported	15	not reported
Katalinic et al. 2013	manuscript	cohort	not reported	39	not reported
Kizony et al. 2016	manuscript	experiment	subacute/chronic	20	not reported
Kurland et al. 2014	manuscript	cohort	chronic	5	1
Lavoie et al. 2016	manuscript	case study	chronic	1	not reported
Mallet et al. 2016 (1)	manuscript	cohort	acute	30	21
Mallet et al. 2016 (2)	abstract	cohort	acute	12	not reported
McCormick & Holmes 2016	abstract	cohort	subacute/chronic	13	not reported
Rand et al. 2013	manuscript	cohort	subacute/chronic	11	not reported
Routhier et al. 2016	manuscript	case study (2)	chronic	2	not reported
Salaris et al. 2016 (1)	abstract	cross-sectional	not reported	63	not reported
Salaris et al. 2016 (2)	abstract	cross-sectional	not reported	15	not reported
Stark & Warburton 2016	manuscript	experiment	chronic	10	3
White et al. 2015	manuscript	qualitative	subacute/chronic	11	0

*Refers to the number of participants with experience using mobile tablet computers prior to their participation in the study.

The majority of studies recruited participants in the chronic or subacute stages of stroke (14 studies), and only 2 studies included acute stroke patients. Participant stroke stage was either not reported or unclear in the remaining 7 studies. Only 8 studies explicitly reported the number of participants who had previous experience with tablets. On average, nearly half of the participants in these studies had previously used a mobile tablet.

### Targets of mobile tablet-based therapy and methods of administration

Study interventions targeted a range of post-stroke deficits and complications: 11 MTBTs included an intervention for communication, 4 for fine-motor skills, 4 for quality of life (although 3 of these were from the same “TnT” study), 3 for cognitive deficits, 2 for deficits targeted by physiotherapy and 1 each for balance and upper extremity function. Therapies tended to be administered using iPad tablets to provide personalized therapy using a unique assortment of commercially available apps for individual participants (Table 2). Therapies were performed in home or inpatient settings and mostly performed independently with no reported assistance from caregivers or clinicians. However, even when therapies were independent, regular contact was often kept with patients through video-conferencing (Katalinic et al. 2013; Kurland et al. 2014), in-person visits (Lavoie et al. 2016; Routhier et al. 2016), or both (White et al. 2016). Certain MTBTs were only performed independently part of the time, with some therapist-assisted use occurring during tele-rehab sessions (Crotty et al. 2014), in-person meetings (Davis & Holzbach 2014; Des Roches et al. 2015; Janssen et al. 2016), and group therapy sessions (Hoover & Carney 2014).

**Table 2:** Characteristics of attempted mobile tablet-based therapies following stroke.

Study	Target	Independent	Setting	Personalized	App(s)
Carabeo et al. 2014	fine-motor skills	yes	inpatient	No	FINDEX
Choi et al. 2016 (1)	communication	yes	home	Yes	iAphasia
Choi et al. 2016 (2)	upper extremity	yes	inpatient	Yes	Mou-Rehab
Crotty et al. 2014	communication/physiotherapy	partially	home	Yes	commercial
Davis & Holzbach 2014	communication	partially	inpatient	unclear	not reported
Des Roches et al. 2015	communication/cognition	partially	clinic/home	Yes	commercial
Hiyamizu et al. 2013	balance	unclear	not reported	unclear	not reported
Hoover & Carney 2014	communication	partially	inpatient	Yes	commercial
Jang & Jang 2016	fine-motor skills	unclear	not reported	No	unnamed
Janssen et al. 2016	quality of life	partially	inpatient/home	unclear	unclear
Katalinic et al. 2013	communication/cognition/fine-motor skills/relaxation	yes	home	yes	commercial
Kizony et al. 2016	fine-motor skills	yes	in/outpatient	No	commercial
Kurland et al. 2014	communication	yes	home	yes	commercial
Lavoie et al. 2016	communication	yes	home	yes	commercial
Mallet et al. 2016 (1)	communication	yes	acute care	yes	commercial
Mallet et al. 2016 (2)	communication/cognition	unclear	acute care	yes	not reported
McCormick & Holmes 2016	physiotherapy	yes	not reported	unclear	SIMULATe
Rand et al. 2013	fine-motor skills	yes	not reported	No	commercial
Routhier et al. 2016	communication	yes	home	yes	commercial
Salaris et al. 2016 (1)	quality of life	partially	inpatient	Unclear	unclear
Salaris et al. 2016 (2)	quality of life	partially	inpatient/home	unclear	unclear
Stark & Warburton 2016	communication	yes	home	yes	commercial
White et al. 2015	quality of life	yes	home	yes	commercial

Studies often personalized their MTBTs to individual patients by assigning different commercial applications based on assessments (Mallet et al. 2016 (1); Mallet et al. 2016 (2)) or perceived needs (Katalinic et al. 2013; White et al. 2015). Therapies using a single commercially available app (Des Roches et al. 2015) or single app developed by the study team (Choi et al. 2016 (1), Choi et al. 2016 (2), Jang & Jang 2016, McCormick & Holmes 2016), personalized therapies by assigning different modules based on assessments. The most frequently used commercially available were apps were Tactus Language Therapy (Hoover & Carney 2014, Katalinic et al. 2013, Mallet et al. 2016 (1); Stark & Warburton 2016; White et al. 2015), Constant Therapy (Des Roches et al. 2015; Hoover & Carney 2014; Mallet et al. 2016 (1)), and Dexteria (Katalinic et al. 2013; Kizony et al. 2016; Rand et al. 2013). Studies of communication MTBTs presented personalized visual stimuli using a variety of apps including Keynote, iBooks, and PowerPoint.

### Barriers to tablet-based care following stroke

Barriers to care, methodological challenges, and patient experience were defined as separate outcomes at the protocol stage but were found to be intertwined during data collection (i.e. barriers to care are often patient reported experiences that lead to methodological challenges). Eight studies reported barriers to MTBT care, which were categorized as device, patient and system barriers (Table 3). The difficulty of the assigned tasks were identified as a device barrier in 3 studies (Carabeo et al. 2014; Choi et al. 2016 (1); Kurland et al. 2014). In each case, participants found the assigned tasks too easy to perform. Bugs in provided videoconferencing software (Katalinic et al. 2013) and the high speed of task prompts and requests (White et al. 2015) were also identified as device barriers. However, the software bug was later resolved.

**Table 3:** Barriers to mobile tablet-based therapy following stroke.

Device Barriers	Patient Barriers	System Barriers
Task difficulty	Difficulty following complex instructions	Unreliable connections
Task bugs	Finger dexterity	Hospital infection control standards
Task speed	Accidently changing crucial settings	Hospital protocol
	Comfort	Network security
	Post stroke depression	
	Strain	
	Patient’s home bandwidth	
	Distractibility	

The most commonly reported patient barrier was a difficulty in following complex instructions (Kurland et al. 2014; Mallet et al. 2016 (1); Routhier et al. 2016; White et al. 2015). Specifically, some participants had difficulty understanding how to use the device (Mallet et al. 2016 (1); White et al. 2015) although it was noted in both cases that participants were able to overcome their difficulties with additional familial support. In other cases, participants had difficulty navigating the device to access the therapy materials (Kurland et al. 2014; Routhier et al. 2016). The second most commonly identified patient barrier was finger dexterity (Kizony et al. 2016; Mallet et al. 2016 (1); Routhier et al. 2016). It was reported that some participant had difficulty isolating their fingers so that only one would touch the screen at a time in order for the device to respond (Kurland et al. 2014), some had difficulty manipulating the device in general, and some had difficulty opening the device case (Mallet et al. 2016 (1)). This final barrier was overcome by using a different tablet case.

Two studies reported patients feeling uncomfortable about specific aspects of their assigned MTBT (Mallet et al. 2016 (1), White et al. 2015). A participant in a MTBT for communication initially felt embarrassed speaking to their device in the hospital, however this was overcome by providing a headset (Mallet et al. 2016 (1)). In another instance, patients felt apprehensive about learning new technology and were particularly anxious about making mistakes in front of study staff (White et al. 2015). These participants felt more comfortable exploring the device at home. Various other patient barriers were reported by single studies. One participant did not use their tablet due to post-stroke depression and feelings of overwhelming post-stroke change (White et al. 2015). Participants taking part in a fine-motor intervention reported the MTBT as being strenuous when performed continually (Carabeo et al. 2014). Particular complains included muscle strain and numbness, hand numbness and jitters, and eye fatigue. Finally, one study reported that the patient’s internet bandwidth made videoconferencing software unusable (Katalinic et al. 2013) and another reported that a participant was particularly excited to use their device for non-therapeutic purposes (Kurland et al. 2014). In the latter case however, this did not appear to interfere with their therapy progress.

System barriers were mostly limited to a study conducted in the acute setting (Mallet et al. 2016 (1)), however two studies of home-based MTBTs also noted barriers in this domain (Katalinic et al. 2013; White et al. 2015). All three of these studies reported connection issues as interfering with care. Some participants lived in areas with poor 3G coverage, resulting in subpar videoconferencing quality (Katalinic et al. 2013). This was resolved by calling participants to help them connect the devices to their home internet connection. However, some participants in another home study reported finding their home internet connections to be unreliable (White et al. 2015). In addition to connection problems, Mallet et al. 2016 (1) also reported barriers related to legitimate concerns from Hospital Infection Control and Hospital IT Security and challenges related to standard of care assessment procedures. Infection control required tablets be cleaned with disinfecting wipes before being transferred to another patient to prevent the spread of disease. There were concerns about hospital network security requiring an independent security assessment before the study could continue. Finally, five eligible patients did not undergo standard of care assessments for unspecified reasons and were missed for recruitment.

Despite these barriers the overall reported patient response was very positive with many studies reporting high satisfaction and usability through either surveys (Choi et al. 2016 (2); Crotty et al. 2014; Kizony et al. 2016; Mallet et al. 2016 (1); Mallet et al. 2016 (2); McCormick & Holmes 2016; Rand et al. 2013) or qualitative interviews (Carabeo et al. 2014; Choi et al. 2016 (1); Hoover & Carney 2014; Kurland et al. 2014; Routhier et al. 2016; White et al. 2015). Specific patient reported positive aspects were the self-directed and independent aspect of tablet-based therapies (Carabeo et al. 2014; Kurland et al. 2014; Routhier et al. 2016; White et al. 2015) and the convenience of home therapy (Crotty et al. 2014; Kurland et al. 2014; Routhier et al. 2016). Patients from three studies indicated that tablet devices were useful rehab tools (Kizony et al. 2016, Rand et al. 2013) and patients from two other studies felt the device contributed to rehabilitation improvements (Carabeo et al. 2014, White et al. 2015). Three studies also reported positive responses from caregivers (Hoover & Carney 2014; Kurland et al. 2014; White et al. 2015) with Kurland et al. (2014) noting that families reported observing improvements and White et al. (2015) indicating that the device gave family and friends an opportunity to engage in therapy with the participants.

### Patient adherence to tablet-based care following stroke

The majority of studies (17/23) prescribed a MTBT dose with only 6 studies not assigning specific MTBT regiments or not reporting this information (Table 4). Prescribed therapy dosages varied although most assigned regiments lasted between 2–6 weeks and assigned 3–6 MTBT sessions a week. Two studies were experiments in which participants only engaged with the MTBT for a single session (Kizony et al. 2016; Rand et al. 2013).

**Table 4:** Therapy dosages.

Study	Assigned Mobile Tablet-Based Therapy Dosage	Clinician-led Sessions
Carabeo et al. 2014	9 sessions of 30 minutes over 1.5 months	standard rehab program
Choi et al. 2016 (1)	4 weeks, as often/long as possible	None
Choi et al. 2016 (2)	10 sessions of 30 mins over 2 weeks	30 minutes of occupational therapy
Crotty et al. 2014	8 weeks	videoconference sessions
Davis & Holzbach 2014	not reported	structured therapy sessions
Des Roches et al. 2015	6 hours a week for 10 weeks plus clinician sessions	10 MTBT sessions (1 per week) over 10 weeks
Hiyamizu et al. 2013	9 sessions (3 per week) over 3 weeks	not reported
Hoover & Carney 2014	20 sessions (5 per week) over 4 weeks	intensive full rehab program
Jang & Jang 2016	24 sessions (6 per week) over 4 weeks	not reported
Janssen et al. 2016	None, could use how they please	standard rehab program
Katalinic et al. 2013	lent device for 3 months	videoconference sessions
Kizony et al. 2016	single use experiment	None
Kurland et al. 2014	120–144 sessions (5–6 per week) over 6 months	24 sessions (1 per week) over 6 months
Lavoie et al. 2016	12 sessions (4 per week) over 3 weeks	weekly non-therapeutic home meetings
Mallet et al. 2016 (1)	1 hour/day during acute stay	standard acute care
Mallet et al. 2016 (2)	1 hour/day during acute stay	standard acute care
McCormick & Holmes 2016	18 sessions over 18 days	not reported
Rand et al. 2013	single use experiment	None
Routhier et al. 2016	20 sessions (4 per week) over 5 weeks	weekly non-therapeutic home meetings
Salaris et al. 2016 (1)	none, could use how they pleased	standard rehab program
Salaris et al. 2016 (2)	none, could use how they pleased	standard rehab program
Stark & Warburton 2016	28 sessions (everyday) for 4 weeks	not reported
White et al. 2015	none, could use how they pleased	Unclear

Tablet-based therapy usage habits were reported by a minority of studies (Table 5). Tablet usage ranged from little daily usage (Kurland et al. 2015) to daily use equal to or exceeding 1 hour (Choi et al. 2016 (1); Mallet et al. 2016 (1); Mallet et al. 2016 (2)). Specific tablet usage was not reported by a handful of studies. In particular, Stark & Warburton (2016) could not confirm usage habits using information transmitted from the tablets and relied on patient self-reported usage. White et al. (2015) qualitatively reported the usage habits of a handful of participants using quotes to communicate the variety of usage habits they observed in their participants.

**Table 5:** Participant mobile tablet-based therapy usage habits.

Study	Mobile Tablet-Based Therapy Usage
Choi et al. 2016 (1)	Mean usage: 60 minutes/day
Des Roches et al. 2015	Mean usage: 40 minutes/day
Kurland et al. 2014	Mean usage: 18 minutes/day
Mallet et al. 2016 (1)	Mean usage: 150 minutes/day
Mallet et al. 2016 (2)	Mean usage: 85 minutes/day
McCormick & Holmes 2016	Participants completed at least 30 minutes of scheduled 90 minute sessions
Salaris et al. 2016 (2)	Used 2–3 times/week during inpatient stay and up to 1–2 hours/days after discharge
Stark & Warburton 2016	Patients reported using table at least 20 minutes every day for 4 weeks
White et al. 2015	Patients reported variable tablet-usage habits

## Discussion

This systematic scoping review yielded 23 eligible articles pertaining to mobile tablet-based therapies for a variety of post-stroke deficits and complications. The majority of the identified articles reported the results of observational studies with small samples of chronic or subacute stroke patients. The goals of most of the studies were exploratory, with only a few articles reporting the results of experimental studies attempting to demonstrate treatment effectiveness. The collected body of literature suggests MTBTs following stroke may be feasible and acceptable, with many studies expressing positive experiences. However, common barriers to care were identified that may prevent certain patients from successfully engaging in therapy.

### Mobile tablet-based communication, cognitive, and fine-motor therapy following stroke

The majority of the attempted MTBTs targeted communication, cognitive, and fine-motor deficits. Considering interventions for these deficits are arguably the most intuitive to implement on a tablet device, it is not entirely surprising that research has focused mainly on these interventions. Furthermore, there is already research supporting the effectiveness of computer-based speech-language therapy [4]. This research-base, coupled with the emergence of popular therapeutic apps like Constant Therapy, Tactus Therapy, and Dexteria may explain why many researchers are more interested in attempting MTBTs for these deficits over other post-stroke complications. Although the methods of administration varied among MTBTs for communication and fine-motor deficits, some trends emerged.

Most MTBTs were performed independently without clinician guidance, although in some cases caregivers or family members participated in therapy with patients. Regular contact with clinicians through videoconferencing and in-person visits were common even among independently performed therapies. The involvement of others in therapy may be beneficial for patient barriers related to therapy and device complexity could be overcome with assistance. Contact with clinicians offers an opportunity for patients to voice issues regarding tablet complexity and for therapists to re-explain complex therapy tasks or features to participants and their caregivers. However, therapists should try not to infringe upon patient independence as this was found to be one of the aspects of tablet-based therapy that patients enjoyed. Similarly, patients would likely prefer for physicians to keep contact in a manner that does not require them to leave their home as the home-based aspect of therapy was also frequently noted as a positive attribute. Videoconferencing was successfully used to contact patients, although some technical difficulties were noted and it is possible that not all patients will have access to reliable cellular or wireless internet connections.

Mobile tablet-based therapies tended to use personalized tasks tailored to patient deficits. Personalization may prevent patients from encountering therapies with inappropriate difficulty levels and encourage extended therapy engagement. However, this relationship between personalization and therapy engagement was not made clear from the collected usage data. It should be noted that even studies with personalized therapy tasks reported barriers to care involving task difficulty suggesting personalization alone is not enough. Rather, therapies should both be personalized and then adjusted as patients engage in therapy. The result is an iterative process whereby patients are assigned tasks based upon their deficits, engage in therapy, express difficulty or make progress, and then the process begins again as new tasks are assigned. This also further highlights the importance of maintaining regular contact with clinicians who are needed to make informed therapy adjustments.

### Mobile tablet-based physiotherapy

Mobility and upper extremity deficits are very common post-stroke [1–2], and yet there were only four studies involving tablet-based physiotherapy activities, two of which were reported as short conference abstracts. Perhaps, the lack of physiotherapy interventions reflects the difficulty of translating traditional physiotherapy to tablets or perhaps due to interests in other promising technology such as robotics [40,41]. Although the few studies reported heterogeneous administrative methods, interesting ideas for tablet-based physiotherapy were described. One intervention for upper extremity therapy used a combination of tablet sensors with a smartphone attached to the patient upper limb to track movement in response to tablet therapy activities which was well-received by patients (Choi et al. 2016 (2)). Other studies used video capabilities to provide visual feedback on movements or provide visual demonstrations (Hiyamizu et al. 2013), and assigned patients a Fitbit to track activity levels (Crotty et al. 2014).

The ability of tablets to host video conferences with therapists could be particularly useful for providing tablet-based physiotherapy. Crotty et al. 2013 reported using a combination of video conferences to deliver therapy, and video recording to save sessions for later viewing, however the details of this administrative method with regards to physiotherapy were not made clear. Further detailed studied of similar administrative approaches and barriers to care would be helpful for determining the feasibility of tablet-based physiotherapy in the home setting. Although patients would likely enjoy the convenience of home physiotherapy instead of travelling to or staying in a rehabilitation center, the safety of home-based physiotherapy is unknown.

### Mobile tablet-based depression treatment

There were no tablet-based mood interventions published, despite depression being common following stroke [42]. However, three abstracts from one study [26,36,37] and one further study [29] reported on interventions targeting quality of life, a construct related to depression. Although the results provide some promise that simply providing patients with a tablet computer could improve quality of life, and therefore mood, it remains unknown if a more structured approach to tablet-based mood therapy is feasible. Interestingly, another study reported improvements in mood and arm function after a physiotherapy intervention [33]. This suggests that providing tablet-based interventions to improve physical deficits could indirectly lead to improvements in post-stroke depression. Logically some patients would feel depressed following the sudden loss of their physical abilities and independence following stroke, and it follows that improvements in physical function could be coupled with an improvement in mood. Future studies of tablet-based intervention targeting physical abilities should consider measuring mood as a secondary outcome to further explore this hypothesis.

Designing and administering tablet-based interventions directly targeting mood is difficult, as no intervention has distinguished itself as particularly effective with stroke patients using a traditional face-to-face administrative approach. For example, although there has been considerable research on internet-based cognitive behavioral therapy in other populations [43,44], it remains unclear if this approach is effective for individuals post-stroke [45]. However, there has been promising evidence for the effectiveness of mindfulness-based therapies for improving mood post-stroke [46] and there exists the possibility of translating these interventions to tablet devices in the future.

### Mobile tablet-based therapy for acute stroke patients

Early rehabilitation initiation is important to maximize recovery of function [6,7], and MTBTs could be used to provide early therapy during the significant amount of downtime experienced by patients in acute care [11]. Despite this, the only two studies attempting MTBTs in the acute stroke setting to provide early stroke rehabilitation services (Mallet et al. 2016 (1); Mallet et al. 2016 (2)) were performed by our own Ottawa Stroke Program Research Team. The technology, need, and available time exist to administer tablet-based therapy in the acute stroke setting. Further investigation into this area is warranted.

### The next steps for mobile tablet-based therapies following stroke

This review provides a summary of MTBT administration methods, barriers to care, and therapy adherence following stroke. The accumulated studies suggest many stroke patients are able to successfully engage in tablet-based therapies for communication, cognitive and fine-motor deficits with minimal assistance and enjoy the independence and convenience of tablet-based therapy despite barriers to care. Attempting randomized controlled trials for therapies targeting communication, cognitive, and fine-motor deficits may not yet be appropriate despite seemingly strong support for treatment feasibility. Further studies should track therapy adherence and experiment with methods for encouraging patient adherence to therapy regiments. Successful methods for promoting therapy engagement may be simple as sending text notifications to the tablet device, setting weekly therapy goals, regular videoconferences, or perhaps a combination of strategies individually tailored to patient preferences.

### Limitations

The large breadth of outcome information eligible for extraction from articles by the two independent reviewers means some relevant outcomes could have been missed. However, the data extraction process used assistive documents to help reviewers identify relevant outcomes. No risk of bias assessment was performed on the included studies as is common for scoping reviews. However, this was done in accordance with current scoping review guidelines and considering the goals of the review were to collect information on administrative methods and barriers to care, a risk of bias assessment would be unlikely to impact our findings.

### Conclusions

The majority of MTBTs following stroke targeted communication, cognitive, and fine-motor deficits, and were positively received by patients despite barriers to care. The findings suggest tablet-based therapy may be feasible for certain stroke deficits although little is known about therapy adherence. Feasibility studies should continue to refine the administrative methods for frequently targeted post-stroke deficits to minimize barriers to care and maximize treatment adherence.

## Supporting information

S1 Checklist PRISMA checklist. (DOC)

S1 Protocol Study protocol. (DOCX)

S1 Appendix Article screening form. (DOCX)

S2 Appendix Data extraction form. (DOCX)
