# Supplementary material for: Mobile tablet-based therapies following stroke: A systematic scoping review of administrative methods and patient experiences
Source: PLoS One. 2018 Jan 23;13(1):e0191566. doi: 10.1371/journal.pone.0191566 (PMC5779660; doi:10.1371/journal.pone.0191566)
Supplement: S1 Protocol — (DOCX) [file pone.0191566.s002.docx]

**Mobile tablet-based therapies following stroke: a systematic scoping review protocol of attempted interventions and the challenges encountered**

This protocol is for an original systematic scoping review, not an update to a previous review.

Authors:

Michael Pugliese (corresponding author), University of Ottawa, School of Epidemiology, Public Health, and Preventive Medicine, Alta Vista Campus, Room 101, 600 Peter Morand Crescent, Ottawa, ON, Canada, K1G 5Z3, [mpugl038@uottawa.ca](mailto:mpugl038@uottawa.ca).

Dylan Johnson, University of Ottawa, School of Epidemiology, Public Health, and Preventive Medicine, Alta Vista Campus, Room 101, 600 Peter Morand Crescent, Ottawa, ON, Canada, K1G 5Z3, djohn051@uottawa.ca.

Dr. Dar Dowlatshahi, Department of Medicine (Neurology), University of Ottawa Brain and Mind Research Institute, and Ottawa Hospital Research Institute, C2182 Ottawa Hospital Civic Campus, 1053 Carling Avenue, Ottawa, ON, Canada, K1Y 4E9, ddowlat@toh.ca.

Dr. Tim Ramsay, University of Ottawa, Ottawa Hospital Research Institute and Scientific Director at the Ottawa Methods Centre, Alta Vista Campus, 501 Smyth Rd, Ottawa, ON, K1H 8L6, tramsay@ohri.ca.

Amendments: amendment to protocol previously registered on January 11^th^, 2017 (<http://hdl.handle.net/10393/35696>). uO Research does not allow authors to host updated protocols. Protocol changes will be tracked and described in the final study manuscript along with justifications.

**ABSTRACT**

**Background:** Stroke survivors suffer from a variety of post-stroke complications and disabilities, diminishing their ability to function independently. Although specialized stroke rehabilitation improves the recovery of functional abilities, accessing rehabilitation services has become increasingly challenging as the absolute number of stroke survivors continues to increase and rehabilitation resources remain scarce. Mobile tablet-based therapies (MTBTs) may provide resource-efficient access to stroke rehabilitation by acting as a platform for therapeutic services. The feasibility and challenges of offering MTBTs to stroke survivors should be well understood before expensive, large-scale clinical trials are undertaken in attempt to demonstrate treatment efficacy.

**Methods:** A systematic scoping review will be conducted to describe attempted MTBTs following stroke, and the challenges encountered by survivors and researchers. Studies of interest will evaluate MTBTs offered to adult stroke patients in response to post-stroke complications or deficits. Journal databases, Grey literature databases, clinical trial registries, relevant organizational websites, and reference lists of eligible studies will be searched to identify eligible studies. Study characteristics, barriers to care, methodological challenges, patient-reported outcomes, and health outcomes will be extracted to describe MTBTs and understand the challenges encountered in context. Results will be presented using descriptive statistics, tables, figures, and a narrative description to summarize the scope of the field.

**Discussion:** Implications for future research will be discussed including research gaps, proposed solutions to common patient and methodological challenges, recommendations for randomized clinical trials of MTBT efficacy, and the appropriateness of conducting a systematic review and meta-analysis of completed pilot trials.

**Systematic review registration:** uO Research (<http://hdl.handle.net/10393/35696>), no registration number given.

**Keywords:** stroke rehabilitation, mHealth, iPad, tablet computer.

**INTRODUCTION**

**Rationale**

*Description of the condition:*

Stroke is the 2^nd^ leading cause of death,^1^ the 3^rd^ leading cause of disability worldwide,^2^ and incurs billions of dollars in healthcare costs annually.^3^ Already enormous, the burden of stroke has continued to increase with a reported 68% rise in the absolute number of first-time incidences of strokes and an 84% increase in stroke survivors in 2010 when compared to 1990.^4^ Stroke survivors experience a wide range of impairments limiting their ability to function independently. Up to two-thirds of survivors experience some form of vascular cognitive impairment,^5^ one-third have communication deficits (aphasia),^6^ 69% have upper limb paralysis,^7^ and 63% are unable to walk independently.^8^ Although the severity of these conditions may improve naturally over time,^9^ symptoms often persist long after patients have been discharged from acute care, and recent studies show a growing number of survivors are struggling to fully recover their pre-stroke level of function.^4,10,11^

Specialized stroke rehabilitation has been shown to effectively improve functional independence following stroke^12^ with the greatest improvements being seen when therapy begins early post-stroke^13,14^ and is performed intensely.^9^ Although the optimal time for beginning stroke rehabilitation is unknown,^15^ initiating therapy 5 to 7 days post-stroke is thought to maximize recovery of functional abilities.^14^ Organized stroke rehabilitation is a multidisciplinary effort involving the cooperation of healthcare professionals from different fields with special expertise in stroke. ^16^ Best practice recommends timely transfer of stroke survivors to well-staffed, specialized inpatient rehabilitation units with sufficient resources to offer the variety and intensity of therapy needed to reduce chances of death and improve functionality.

*Description of the problem:*

Accessing timely rehabilitation is challenging with more than 50% of Canadians beginning stroke rehabilitation after 13 or more days.^17^ Similar trends are seen in the United States where only 24% of patients are transferred to inpatient services and wait an average of 27 days post-stroke before beginning rehabilitation.^18^ Evidence suggest patients only spend 13% of their time in acute care engaging in activities to improve their recovery meaning many patients are not initiating intense therapy in a timely manner.^19^ Furthermore, only 16% of stroke survivors are eventually transferred to inpatient rehabilitation centers when the 40% of survivors would benefit.^17^ Without access to inpatient services, survivors are forced to seek outpatient care. However, accessing outpatient care is also difficult,^20^ with the number of survivors reported to be receiving outpatient services being lower than expected.^21^

Poor access to rehabilitation services has been speculated to be caused by a lack of resources, namely therapists with expertise in stroke, in the acute,^19^ inpatient ^17^ and outpatient settings.^20,21^ There is evidence to support this speculation with therapist-bed ratios in Ontario rehabilitation centers ranging from 10.5 to 56 beds per therapist .^22^ Beds themselves are also scarce with only 270 known dedicated stroke beds in 54 centers. With the absolute numbers of stroke survivors increasing worldwide, creative and cost-effective solutions are needed to improve timely access to intense stroke rehabilitation. Otherwise, judging by current resources, more stroke survivors will be unable to begin timely and intense therapy to maximize their recovery and function independently.

*The proposed solution:*

Over the past decade there has been growing interest in harnessing technology to support stroke rehabilitation and provide therapy. Recent technological advances in smart-technology have made smartphones and mobile tablet-devices practically ubiquitous with a vast array of software applications (apps) available either for free or inexpensively via online app stores. Accordingly, there has been growing interest in exploring the possibilities offered by these devices with particular interest in tablet-computers as platforms for therapy.

Mobile tablet-based therapies (MTBTs) use apps running on tablet computers to provide interventions to patients. There are a variety of potentially therapeutic apps including those explicitly designed to offer therapy (Constant Therapy for aphasia and cognitive impairments) and apps offering activities analogous to scenarios often used in stroke rehabilitation (memory and attention games, etc.). Survivors could use MTBTs to begin therapy in acute care, supplement inpatient or outpatients care if therapists are not available, or provide their sole source of therapy if no other services are accessible.

## Why is it important to do a scoping review now?

Despite their wide availability, relatively low cost, and technological power, much remains unknown about MTBTs including treatment efficacy. However, before attempting small or large-scale trials of treatment efficacy, the feasibility and challenges of offering MTBTs following stroke should be well understood in order to improve the chances of conducting successful trials. Therefore, a systematic scoping review is needed to answer key questions regarding MTBTs following stroke in order to confidently move towards conducting methodologically robust studies of treatment efficacy.

**Objectives:** To review the evidence for mobile tablet-based therapies (MTBTs) following stroke.

*Research questions:*

1. What are the characteristics of attempted MTBTs following stroke in terms of deficits targeted and method of administration?
2. What barriers or adverse events related to the administration of MTBTs following stroke have been encountered by researchers, clinicians, caregivers, or participants?
3. What methodological challenges have been faced by studies of MTBTs following stroke?

**METHODOLOGY**

There is currently no standard, widely accepted guidance document equivalent to PRISMA-P for the development of scoping review protocols. This protocol was developed primarily with the assistance of a current published guideline for scoping reviews^23^ and with PRISMA-P when necessary and appropriate to design a robust and systematic scoping review.^24^

**Inclusion/Exclusion Criteria (summary):**

Inclusion criteria (must meet all):

1. Adult stroke survivors (18 years or older) of any type (ischemic/hemorrhagic) or stage (acute/chronic) in any setting.
2. Stroke survivors interacting with a mobile tablet in response to a post-stroke deficit or complication for therapeutic purposes.

Exclusion criteria (exclude it meet one or more):

1. The mobile tablet is primarily used by someone other than the stroke survivor.
2. The mobile tablet is used primarily for screening, assessment, data collection, or is not the primary therapeutic device.

**Criteria Explanation and Elaboration:**

*Population:*

We are only including adult stroke survivors; children are a separate population outside of the scope of the proposed review. There are no restrictions with regard to stroke type or stage as the field is expected to be heterogeneous in this regard. Studies involving a mixture of stroke and non-stroke participants will be included and information will only be extracted for stroke participants if possible.

*Intervention:*

We define MTBTs as patient-driven therapies where participants interact via any modality with mobile tablet devices in response to any deficit or complication where the tablet device is the primary method of therapy delivery. Therapies involving peripheral devices (smartphone, robotics, sensors, etc.) will be included if the mobile tablet is clearly the primary device for delivering therapy.

The use of tablets as assistive devices by clinicians for the administration of therapy, or by patients for the primary purpose of screening, assessment, or data collection does not constitute a MTBT. Tele-rehab programs using tablets solely as a method of videoconferencing with participants will not be included as the therapy is not truly mobile tablet-based; the tablet is simply acting as a means of providing traditional treatment.

*Context:*

There are no restrictions related to context as we are interested in interventions performed in all settings and geographical locations, administered in all languages, and delivered by all types of therapists or non-therapists.

*Comparator(s):*

There are no restrictions related to comparators as we are interested in describing all attempted MTBTs following stroke regardless of comparisons to other therapies.

*Outcomes:*

There are no restrictions with regards to study outcomes as we are primarily interested in attempted interventions, therapy barriers, and research challenges. However, considering the study goals and research questions, we are interested in study outcomes including but not limited to: barriers to care, adverse events, protocol deviations, Research Ethics Board issues, recruitment rate, adherence rate, retention rate, and patient evaluations of MTBTs.

*Study designs:*

There are no restrictions with regards to study design: observational and experimental studies of all designs will be included. There will be no restrictions with regards to study timing as it is expected that studies will substantially vary in length and timing. Study protocols and conference abstracts will only be included if they contain pilot or preliminary results from a study whose data are otherwise unavailable from a full study manuscript.

*Other restrictions:*

The included studies will be restricted to those written in English due to translation costs. Although the first modern tablet computers were introduced in the early 2000s, the surge in popularity of tablet computers with the release of the first Apple iPad in 2010 is well known. Therefore, searches will be restricted to include studies between 2010 to present.

**Information Sources**

*Preliminary search:*

A preliminary search of the literature using key terms related to stroke and mobile devices in MEDLINE (OVID interface) yielded a number of studies meeting our inclusion criteria. These articles were used to identify key words and build a search strategy with the aid of a health information librarian. One study author (MP) piloted the search strategy in MEDLINE to ensure the strategy successfully re-identified the studies used to build the search. The search strategy successfully identified these papers and no further modifications were made to the strategy except those necessary to adapt the strategy to different database search interfaces.

*Database searches:*

The following six databases will be searched: MEDLINE (OVID interface), EMBASE (OVID interface), PsycINFO (OVID interface), CINAHL, Cochrane Database, and Web of Science.

*Additional information sources:*

1. A snowball search of relevant articles and reviews identified by the database search.
2. Organizational websites: Aphasia.org, American Stroke Association webpage, Heart and Stroke Foundation webpage, and Stroke Engine.
3. Clinical trial databases will also be searched for completed and ongoing studies: ClinialTrials.gov, the WHO International Clinical Trials Registry Platform, EU clinical trials database and ISRCTN.

*Grey literature search:*

A grey literature search will also be performed in order to find unpublished material using Google Scholar, the ProQuest Dissertation and Theses Database (Global and UK & Ireland), and the OpenGrey European grey literature database. After a preliminary search of Google Scholar and ProQuest Dissertation Global it was decided searches would be limited to the first 200 results as a compromise between conducting a robust search and exhausting resources as search results beyond the first 200 results appeared to be irrelevant.^25^

**Database Search Strategy**

The search strategy presented below was used to search databases with Ovid interfaces and adapted to search databases without an Ovid interface. All index database searches were restricted to between the years 2010 – present and English language.

*Medline (Ovid interface) search strategy.*

1. exp Stroke/

2. exp cerebrovascular disorders/

3. (stroke* or cerebrovascular* or cerebral vascular or CVA*).tw.

4. ((cerebr* or brain) adj3 infarct*).tw.

5. 1 or 2 or 3 or 4

6. (mobile device* or mobile computer* or handheld computer* or tablet*).tw.

7. (ipad* or galaxy tab* or surface pro*).tw.

8. 6 or 7

9. 5 and 8

**Study Records**

*Data management*

Database search results will be downloaded and imported to reference management software (Endnote X8) in order to search for duplicates.^26^ After duplicates have been removed using both software and manual identification, the database results will be uploaded into Covidence, an online systematic review manager, where all abstract screening and full-text reviewing will take place.^27^

*Selection process*

Two authors (MP and DJ) will independently screen collected articles in a two-stage process with the assistance of an article screening form (Appendix A): (1) co-screeners will review study titles and abstracts returned by database searches for potentially eligible studies, and (2) screen full-text manuscripts to confirm eligibility. The screening form will be piloted on batches of 30 and refinements to the form made until an inter-rater agreement, as measured by the Kappa statistic, of .80 or above is achieved. ^28^ Screening conflicts will be resolved through discussion between screeners or resolved by a third party (DD) if necessary during both stages of screening. Reasons for full-text study exclusion will be tracked and listed. Screeners will not be blinded to study authors, affiliated institutions, or journal titles.

*Data collection process*

Two authors (MP and DJ) will independently extract key data items needed to describe the included studies and to answer the stated research questions. A data extraction form (Appendix B) will be used to guide data collection. Authors will compare and consolidate extracted information regularly to create a final data extraction form for each study. As the extraction process progresses, the data extraction form will be refined as necessary and any new pieces of information not collected in studies screened before changes occurred will be obtained. Study outcomes will be classified into one of four categories: barriers and adverse events, methodological challenges, patient reported outcomes (non-health), and health outcomes. An assistive document (Appendix C) will be used to help with the categorization of outcomes. Data extraction conflicts will be resolved through discussion between screeners or resolved by a third party (DD) if necessary. No effort will be made to collect missing information from study authors due to time constraints.

**Data Items**

A wide variety of data items are needed to adequately answer the proposed research questions in context. Although every effort has been made to anticipate the broad extent of variables which will be collected, refinements to collected information will be made as the data collection process progresses if deemed necessary. Data items of interest fall into 6 categories: general study information, participant characteristics, intervention details, comparator details, outcomes, and setting and context. A full list of variables and clarifications where necessary can found in Appendix B.

**Outcomes and Prioritization**

There is no need to prioritize outcomes to accomplish the objective of the review. All data items and outcomes of interest will be reported, discussed and interpreted holistically to answer research questions.

## **Risk of Bias in Individual Studies**

As per current systematic scoping review guidelines, no formal risk of bias assessment will be performed for the included studies.^23^ However, the potential impact of study design on individual study results will be discussed as the collected data items allows for an informed commentary.

**Data**

*Presentation of the results*

Determining how to best present the results of a scoping review is an iterative process where the most logical approach becomes clearer as the data charting process comes closer to being completed. Therefore the outline below is expected to be refined throughout the data extraction process.

Search results will be summarized narratively and using the PRISMA flow diagram (Figure 1).^29^ Key study characteristics, and information related to review questions will be summarized narratively, using descriptive statistics, and in table or figures as necessary. Health and patient reported outcomes will be reported, however no attempt to quantitatively synthesis results will be attempted due to anticipated study heterogeneity.


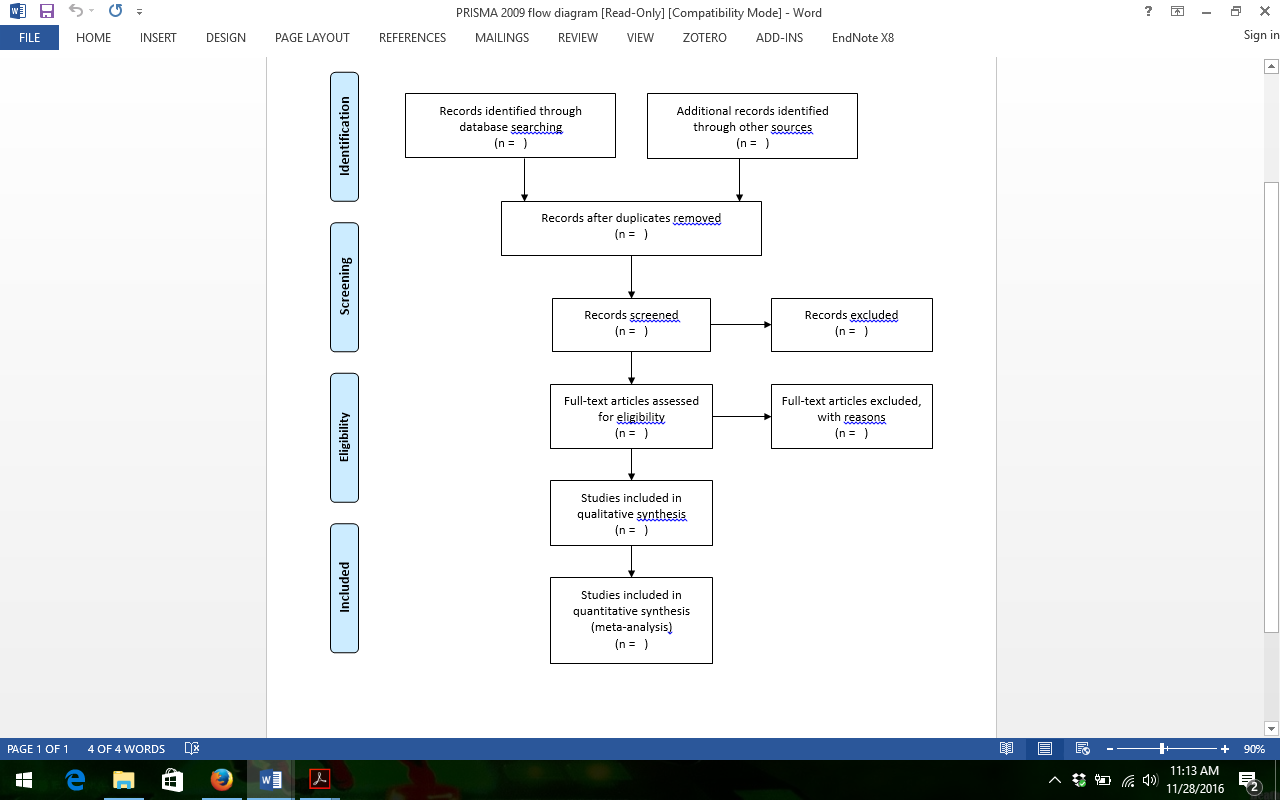


**Figure 1. PRISMA 2009 flow diagram.**

**Meta-Bias(es) and Confidence in the Cumulative Evidence**

There is no planned formal assessment of meta-biases or the confidence in the accumulated body of evidence. Instead, the impact of bias and the strength of the evidence will be discussed based on an informal interpretation of the results.

**DISUCSSION**

Answers to study questions will be discussed or if answers are not found, reasons will be discussed. Solutions to common challenges faced by patients and researchers will be proposed and if appropriate, recommendations for evaluating these solutions. Limitations of the reviewed studies and the need for more robust studies in researched areas will be discussed. Gaps in research with special attention to under-studied interventional areas will be discussed and to a lesser extent, under-studied patient populations and settings. Lastly, recommendations will be made for small and large-scale randomized clinical trials of MTBT efficacy, and a comment on the appropriateness of conducting a systematic review and meta-analysis of completed pilot trials.

**LIST OF ABBREVIATIONS**

MTBT: mobile tablet-based therapy

**DECLARATIONS**

**Ethics Approval and Consent to Participate**

Not applicable.

**Consent for Publication**

Not applicable.

**Availability of Data and Materials**

All data generated or analyzed during this study will be reported in the final published manuscript and its supplementary information files.

**Competing Interests**

None.

**Funding**

Mr. Pugliese is supported by an Ontario Graduate Scholarship and Canadian Institutes of Health Research Graduate Scholarship.

Dr. Dowlatshahi is supported by a Heart and Stroke Foundation New Investigator Award and a University of Ottawa Department of Medicine Clinician-Scientist Chair.

No other sources of funding nor sponsors supported the current review. The funding sources had no involvement in developing the study protocol.

**Author’s Contributions**

MP developed study protocol and performed preliminary searches.

DJ developed study protocol.

DD developed study protocol.

TR developed study protocol.

All authors read and approved the final manuscript.

**Acknowledgements**

The authors would like to thank Mish Boutlet of the University of Ottawa Health Sciences library for assisting with the refinement of the database search strategy.

**Author’s Information (optional).**

None.

**RFERENCES**

1. Lozano R, Naghavi M, Foreman K, et al. Global and regional mortality from 235 causes of death for 20 age groups in 1990 and 2010: a systematic analysis for the Global Burden of Disease Study 2010. *Lancet.* 2010;380:2095-2128. doi:10.1016/S0140-6736(12)61728-0.
2. Murray CJL, Vos T, Lozano R, et al. Disability-adjusted life years (DALYs) for 291 diseases and injuries in 21 regions, 1990 – 2010: a systematic analysis for the Global Burden of Disease Study 2010. *Lancet*. 2012;380:1990-2010. doi:10.1016/S0140-6736(12)61689-4.
3. Di Carlo A. Human and economic burden of stroke. *Age Ageing*. 2009;38:4-5. doi:10.1093/ageing/afn282.
4. Feigin VL, Forouzanfar MH, Krishnamurthi R, et al. Global and regional burden of stroke during 1990-2010: findings from the Global Burden of Disease Study 2010. *Lancet.* 2014;383:245-255. http://dx.doi.org/10.1016/S0140-6736(13)61953-4.
5. Salter K, Teasell R, Foley N, Allen L. Evidence based systematic review of stroke rehabilitation: Cognitive disorders and apraxia. Last updated July 2013. Retrieved from: <http://www.ebrsr.com/evidence-review/12-cognitive-disorders-and-apraxia>.
6. Salter K, Teasell R, Foley N, Allen L. Evidence based systematic review of stroke rehabilitation: Aphasia. Last updated September 2013. Retrieved from: <http://www.ebrsr.com/evidence-review/14-aphasia>.
7. Nakayama H, Jorgensen HS, Raaschou HO, Olsen TS. Recovery of Upper Extremity Function in Stroke Patients: The Copenhagen Stroke Study. *Arch Phys Med Rehabil.* 1994;75;394-398.
8. Jorgensen HS, Nakayama H, Raaschou HO, Olsen TS. Recovery of Walking Function in Stroke Patients: The Copenhagen Stroke Study. *Arch Phys Med Rehabil.* 1995;76;27-32.
9. Teasell R, Hussein N. Evidence based systematic review of stroke rehabilitation: Background concepts in stroke rehabilitation. Last updated November 2013. Retrieved from:
10. Stroke report 2015: Access to Stroke Care: The Critical First Hours. Retrieved from: http://www.heartandstroke.com/atf/cf/%7B99452d8b-e7f1-4bd6-a57d-b136ce6c95bf%7D/HSF_2015_STROKE_REPORT_FINAL.PDF. Accessed August 29th, 2016.
11. Krueger H, Koot J, Hall RE, et al. Prevalence of individuals experiencing the effects of stroke in Canada: Trends and projections. Stroke. 2015;46:2226-2231. doi: 10.1161/STROKEAHA.115.009616.
12. Foley N, Teasell R, Bhogal S, Speechley M, Hussein N. Evidence based systematic review of stroke rehabilitation: The efficacy of stroke rehabilitation. Last updated: November 2013. Retrieved from: http://www.ebrsr.com/evidence-review/5-efficacy-stroke-rehabilitation.
13. Cifu D, Stewart D. Factors affecting functional outcome after stroke: a critical review of rehabilitation interventions. *Arch Phys Med Rehab.* 1999;80:S35-39.
14. Ottenbacher KJ, Jannell S. The results of clinical trials in stroke rehabilitation research. *Arch Neurol.* 1993;50(1):37-44. doi: 10.1001/archneur.1993.00540010033014.
15. The AVERT Trial Collaboration group. Efficacy and safety of very early mobilization within 24 h of stroke onset (AVERT): a randomized controlled trial. *Lancet.* 2015;386:46–55. Published online: <http://dx.doi.org/10.1016/S0140-6736(15)60690-0>
16. Heart and Stroke Foundation Canadian Best Practice Recommendations 2016. Stroke Rehabilitation Unit Care. <http://www.strokebestpractices.ca/index.php/stroke-rehabilitation/stroke-rehabilitation-unit-care-2/>. Posted February 2016. Accessed January 22, 2017.
17. Heart and Stroke Foundation 2014 stroke report. Heart and Stroke Foundation website. <http://www.heartandstroke.com/site/apps/nlnet/content2.aspx?c=ikIQLcMWJtE&b=7498307&ct=13940921>. Posted June 2014. Accessed February 2, 2016.
18. Wang H, Camicia M, Terdiman J, Hung YY, Sandel ME. Time to inpatient rehabilitation admission and functional outcomes of stroke patients. *Am J Phys Med Rehabil.* 2011;3:296-304. DOI: 10.1016/j.pmrj.2010.12.018
19. Bernhardt J, Dewey H, Thrift A, Donnan G. Inactive and alone: physical activity within the first 14 days of acute stroke unit care. *Stroke*. 2004;35:1005-1009. DOI: 10.1161/01.STR.0000120727.40792.40.
20. Consensus Panel on the Stroke Rehabilitation System. Time is Function: A report from the Consensus Panel on the Stroke Rehabilitation System to the Ministry of Health and Long-Term Care. *Heart and Stroke Foundation of Ontario*. Published April 30, 2007. Retrieved from: <http://www.heartandstroke.on.ca/atf/cf/%7B33C6FA68-B56B-4760-ABC6-D85B2D02EE71%7D/SRSCP_FULL_REPORT_FINAL20070430%5B1%5D.pdf>
21. Xie J, George M, Ayala C, McGruder H, Denny C, Croft J. Outpatient Rehabilitation Among Stroke Survivors --- 21 States and the District of Columbia, 2005. *Morbity Mortal Wkly Rep*. 2007;56(20):504-507.
22. Meyer M, Foley N, Pereira S, Salter K, Teasell R. Organized Stroke Rehabilitation in Canada: Redefining Our Objectives. *Top Stroke Rehabil*. 2012;19(2):149-157. doi:10.1310/tsr1902-149.
23. Peters MDJ, Godfrey CM, Khalil H, Mcinerney P, Parker D, Soares CB. Guidance for conducting systematic scoping reviews*. Int J Evid Based Healthc*. 2015;13:141-146. doi:10.1097/XEB.0000000000000050.
24. Moher D, Shamseer L, Clarke M, et al. Preferred Reporting Items for Systematic Review and Meta-Analysis Protocols (PRISMA-P) 2015 statement*. Syst Rev*. 2015;4(1):1. doi: 10.1186/2046-4053-4-1.
25. Stevinson C, Lalor DA. Searching multiple databases for systematic reviews: added value or diminishing returns? *Complement Ther Med.* 2004;12:228-232. doi:10.1016/j.ctim.2004.09.003
26. Endnote X8 Reference Management Software. Clarivate Analytics, Copyright 2017 Thomson Reuters. Retrieved from: <http://endnote.com/>.
27. Covidence systematic review online management tool. Teamsquare, Level 2, 520 Bourke Street, Melbourne, Victoria, 3004. Accessible from: <https://www.covidence.org/>.
28. McHugh ML. Interrater reliability: the kappa statistic. *Biochem Med*. 2012;22(3):276-82.
29. Moher D, Liberati A, Tetzlaff J, Altman DG, The PRISMA Group. Preferred Reporting Items for Systematic Reviews and Meta-Analyses: The PRISMA Statement. *PLoS Med*. 2009 6(7): e1000097. doi:10.1371/journal.pmed1000097.

**Appendix A: Article Screening Form**

| **PICOS Element** | **Meets Criteria?** | | **Reason For Exclusion** |
| --- | --- | --- | --- |
| **Population**: Does the study enroll a population of human adults with stroke? | Yes  Unclear | No | 1. Not an adult population.  2. Not a stroke population. |
| **Intervention:** Does the study involve stroke patients interacting with a mobile tablet device in response to a post-stroke deficit or complication? | Yes  Unclear | No | 3. Not a tablet-based therapy.  4. Patient are not the primary tablet users |
| **Study Design:** Does the manuscript report the results of a study? If a study protocol or conference abstract, does it report the results of a study whose data is not otherwise available in a study manuscript? | Yes  Unclear | No | 5. Manuscript is a protocol/conference abstract with data available from a study manuscript. |

**Appendix B: Data Extraction Form**

| **General Study Information** | |
| --- | --- |
| **Study Citation** |  |
| **Purpose/objective** |  |
| **Population** |  |
| **Study Design** |  |
| **Outcome ascertainment (self-report, interview, etc.)** |  |
| **Participants** | |
| **Inclusion/exclusion criteria** |  |
| **Sample Size** |  |
| **Stroke stage (Acute, chronic, mixed, or unknown)** | **Use time from stroke if it’s available, otherwise note stage.** |
| **Stroke type (Ischemic, hemorrhagic, mixed, unknown).** |  |
| **Average/Median age (SD/range/IQR)** |  |
| **Number/% female/male** |  |
| **Stroke severity (Include NIHSS or other measures if given)** |  |
| **Average/Median Time Post-Stroke (SD/range/IQR)** | **Time from stroke onset to initiation of MTBT (or if not explicitly stated, report time to MTBT study enrolment and make note of this).** |
| **Education Level** |  |
| **Number/% Familiar with touch-screen devices** |  |
| **Computer skill** |  |
| **Intervention** | |
| **Device make and model.** |  |
| **Therapy target (speech, fine-motor, etc.).** |  |
| **Therapy completed independently? If not, with whose assistance/oversight?** |  |
| **Used preexisting applications/software? It not, what was used and where did they originate from?** |  |
| **Dose/scheduling** |  |
| **Other important details regarding therapy approach and format**. |  |
| **Comparator(s)** | |
| **Description** |  |
| **Dose/schedule** |  |
| **Outcomes** | |
| **Barriers and Adverse events** |  |
| **Methodological Challenges** |  |
| **Patient Reported Outcomes** |  |
| **Health outcomes** |  |
| **Setting and Context** | |
| **Therapy setting** |  |
| **Recruitment setting (if different from therapy setting)** |  |
| **Geographical location** |  |
| **Language** |  |

**Appendix C: Anticipated Outcome Categories of Included Studies**

| **Outcome Categorization** | |
| --- | --- |
| **Outcome Category** | **Example Outcomes** |
| Barriers and Adverse events | Patient barriers, Device barriers, Environment barriers, Solutions to barriers (proposed/attempted, and success if attempted), Adverse events, Other barriers or possible adverse events |
| Methodological Challenges | Recruitment Rate, Adherence Rate, Retention Rate (loss to follow-up), Reasons for drop-out, Reasons for non-Adherence, Protocol deviations and/or revisions |
| Patient Reported Outcomes | Ratings of perceived usefulness of intervention, Ratings of intervention likability, Other patient opinions. |
| Health outcomes | Global disability, Cognitive impairment, Language impairment, Fine-motor skills, Mobility, Depression. |
