# Supplementary material for: Mobile tablet-based therapies following stroke: A systematic scoping review of administrative methods and patient experiences
Source: PLoS One. 2018 Jan 23;13(1):e0191566. doi: 10.1371/journal.pone.0191566 (PMC5779660; doi:10.1371/journal.pone.0191566)
Supplement: S1 Appendix — (DOCX) [file pone.0191566.s003.docx]

| **PICOS Element** | **Meets Criteria?** | | **Reason For Exclusion** |
| --- | --- | --- | --- |
| **Population**: Does the study enroll a population of human adults with stroke? | Yes  Unclear | No | 1. Not an adult population.  2. Not a stroke population. |
| **Intervention:** Does the study involve stroke patients interacting with a mobile tablet device in response to a post-stroke deficit or complication? | Yes  Unclear | No | 3. Not a tablet-based therapy.  4. Patient are not the primary tablet users |
| **Study Design:** Does the manuscript report the results of a study? If a study protocol or conference abstract, does it report the results of a study whose data is not otherwise available in a study manuscript? | Yes  Unclear | No | 5. Manuscript is a protocol/conference abstract with data available from a study manuscript. |
